# Novolac/Phenol-Containing Phthalonitrile Blends: Curing Characteristics and Composite Mechanical Properties

**DOI:** 10.3390/polym12010126

**Published:** 2020-01-05

**Authors:** Hanqi Zhang, Bing Wang, Yanna Wang, Heng Zhou

**Affiliations:** 1 National Key Laboratory of Science and Technology on Advanced Composites in Special Environments, Harbin Institute of Technology, Harbin 150001, China; zhanghanqi_hit@163.com (H.Z.); wyn191010@163.com (Y.W.); 2 Laboratory of Advanced Polymer Material, Institute of Chemistry, Chinese Academy of Sciences, Beijing 100190, China

**Keywords:** blends, phthalonitrile, composites, curing characterizes, mechanical properties

## Abstract

The phenol-containing phthalonitrile resin is a kind of self-curing phthalonitrile resin with high-temperature resistance and excellent properties. However, the onefold phthalonitrile resin is unattainable to cured completely, and the brittleness of the cured product is non-negligible. This paper focuses on solving the above problems by blending novolac resin into phenol-containing phthalonitrile. Under the action of abundant hydroxyl group, the initial curing temperature and gelation time at 170 °C decrease by 88 °C and 2820 s, respectively, monitored by DSC and rheological analysis. FT-IR spectra of copolymers showed that the addition of novolac increased the conversion rate of nitrile. When the novolac mass fraction is 10%, the peak of nitrile group disappears, which means the complete reaction. The mechanical test of blends composites shows that the maximum fracture strain of 10 wt% novolac addition is 122% higher than those of neat phthalonitrile composites on account of the introduction of flexible novolac chain segments. The mechanical properties are sensitive to elevated post-cured temperature; this is consistent with the result of morphological investigation using SEM. Finally, the dynamic mechanical analysis indicated that the glass transition temperature heightened with the increase of novolac content and post-curing temperature.

## 1. Introduction

The advanced polymeric composites are required to have a serious of performance like excellent mechanical properties, superior heat resistance, and certain functionality. The distinguished comprehensive performance of these composites is conductive for pervasive application in marine, aerospace, radiation shielding, and electronic fields [1,2,3,4,5]. The common resin matrix for advanced composites are epoxy [6,7], polyimide [8,9,10], polyetheretherketone [11,12], and the best heat-resistant resin during last four decades—phthalonitrile [13,14,15,16,17].

Phthalonitrile resin possesses excellent mechanical properties, high glass transition temperature, superior moisture, and outstanding thermal stability due to their highly aromatic and heterocyclic ring structures [18]. However, the formation of heterocyclic ring by nitrile nucleophilic substitution is unusually slow, and the conversion is limited [19]. Previous researchers designed and synthesized a wide variety of phthalonitrile monomers to alleviate the problem [20,21,22]. The curing reaction of these phthalonitrile monomers was assisted by a series of curing agents, such as transition metal, transition metal salts [23], organic amine [24], and phenols [25]. Nevertheless, the vast majority of the curing agent has inferior thermal stability. The decomposition of the curing agent at elevated temperature during the consolidation results in internal defects that will affect the structure integrity of the system. In this case, the widely accepted strategy is to design the self-curing phthalonitrile [26,27,28,29,30].

The phenol-containing phthalonitrile was an attractive kind of self-curing phthalonitrile, synthesized by condensation of novolac with 4-nitrophthalontrile [31,32,33,34]. The hydroxyl group of nucleophilic phenol groups in the phenol-containing phthalonitrile skeleton offered a rapid proton to catalyze the reaction of the nitrile groups in the same skeleton [18]. However, the steric hindrance effect limited the obtainment of the fully cured polymer, and the brittleness problem of phthalonitrile lacked a proper solution [35].

In the present work, as an additive, the novolac was added to phenol-containing phthalonitrile resins, with the purpose of improving the processability and curing degree via increasing the phenolic hydroxyl concentration. Furthermore, the introduction of flexible phenolic chain segments was expected to overcome the intrinsic brittleness of polymer. Different compositions of novolac/phthalonitrile blends and copolymers were prepared. The curing properties, structure transition and thermal oxygen stability affected by novolac content were investigated by DSC, rheological experiment, FT-IR and thermogravimetric analysis. Besides, we also conducted the novolac/phthalonitrile-carbon fiber fabric composites with different novolac mass percent and post cured at different processes. The mechanical properties of composites were obtained by three-point bending test and uniaxial tensile experiment, and the fracture surface was observed using SEM to find the evidence of toughening. Finally, the glass transition temperature of composites was measured by dynamic mechanical analysis.

## 2. Materials and Methods

### 2.1. Materials

**Figure 1:**
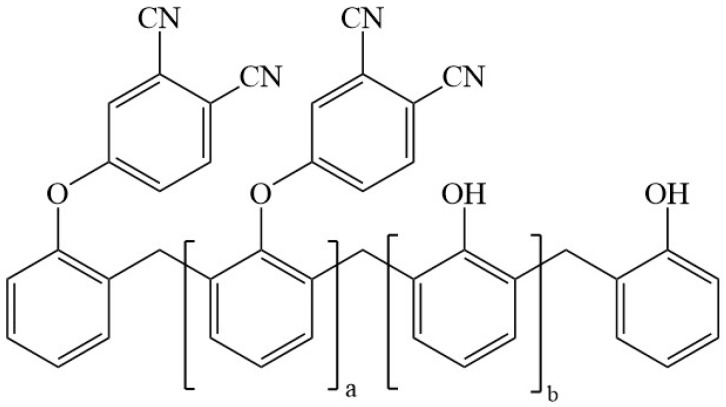
Structure of phenol-containing phthalonitrile monomer [36].

The phenol-containing phthalonitrile monomer was obtained from Institute of Chemistry, Chinese Academy of Sciences (Beijing, China) [16,36]. Novolac resin (molecular weight: 2000) was purchased from GuangZhou Liben Rubber Raw Materials Co., Ltd (Guangzhou, China). Carbon fiber fabric (3K-1000) was provided by Japan Toray Co., Ltd (Tokyo, Japan). The structure of phenol-containing phthalonitrile monomer is shown in Figure 1.

### 2.2. Preparation of Novolac/Phenol-Containing Phthalonitrile Blends and Copolymers

Novolac resin and butanone solution with the weight ratio as 50:50 were put in the same beaker. The mixture was stirred at a constant speed at 50 °C for 10 min until it became uniform transparent liquid. In a three-mouth flask equipped with mechanical stirrer, heater, and thermometer, phthalonitrile resin was heated into melt at 70 °C, and then novolac/butanone mixture was added with constant stirring for 60 min to achieve a homogeneous compound. Considering that the boiling point of butanone is 78 °C, the temperature of the compound was raised to 85 °C for 15 min to remove the residual solvent in the mixture. According to different additive weight of novolac, 5% and 10% mass percent novolac/phenol-containing phthalonitrile blends were obtained. Neat phthalonitrile polymers and those containing novolac at 5 and 10 wt% were cured at 200 °C (1 h), 260 °C (4 h), and 310 °C (5 h). To clarify the relationship between the sample name and composition, the neat phenol-containing phthalonitrile resin is abbreviated PN. The blends are named PF 5% and PF 10% according to their novolac mass fraction of 5% and 10%. All cured resins are prefixed with a “c” before the abbreviation, like cPN, cPF 5% and cPF 10%.

### 2.3. Preparation of Carbon Fabric Reinforced Novolac/Phenol-Containing Phthalonitrile Prepregs and Composites

Novolac/phenol-containing phthalonitrile-carbon fabric prepreg was formulated by hot-press process. Carbon fiber fabric was cut into 30 mm × 30 mm, and then heated novolac/phenol-containing phthalonitrile blends were evenly applied to the surface of the fabric at 120 °C. The impregnated fabric was transferred to oven, and then pressure by counterweight was applied in the continuous condition of 120 °C for 30 min. After that process, the fabric was quenched to room temperature to form a prepreg. Compression molding technique was used to prepare the novolac/phenol-containing phthalonitrile-carbon fiber fabric composite on the thermopress. The detailed curing procedures of composites are exhibited in Table 1. The composite samples are named according to their resin composition, such as the composite consists of blend containing 5 wt% novolac is named PF 5%.

### 2.4. Characterization

Differential scanning calorimetric (DSC) experiments on the novolac/phenol-containing phthalonitrile blends and copolymers were performed, using a Netzsch STA449F3 calorimeter (Netzsch, Selb, Germany) at a heating rate of 10 °C/min in a nitrogen atmosphere between 25 °C and 450 °C. Rheological behavior of curing process was determined by TA Instruments DHR-2 (TA Instrument, Boston, Massachusetts, US) at low strain values (0.5%) with a 25-mm parallel plate fixture and 1 Hz frequency in air environments. Fourier transform infrared spectroscopy (FT-IR) spectra were recorded on a Nicolet Is50 (Thermo Scientific, Billerica, Massachusetts, US) using KBr pellets between 4000 and 400 cm^−1^. Thermal oxygen stability of the copolymers was observed, using a Netzsch STA449F3 (Netzsch, Selb, Germany) at a heating rate of 10 °C/min in a rage of 35 to 800 °C under the flowing of air.

Dynamic mechanical analysis (DMA) of novolac/phenol-containing phthalonitrile-carbon fiber fabric composites were conducted by a TA Instruments Q-800 (TA Instrument, Boston, Massachusetts, US) at a heating rate of 5 °C/min with 1 Hz frequency in nitrogen atmosphere. The microstructure of fracture surface of composites was studied using a scanning electron microscope (Sirion 200, FEI, Hillsboro, Oregon, US). Mechanical properties of the composites were measured by an INSTRON 5569 (Instron, Norwood, Massachusetts, US) at room temperature. Tensile properties and flexural properties were tested according to ASTM D3039 and ASTM D7264, respectively.

## 3. Results and Discussion

### 3.1. Curing Properties of Novolac/Phenol-Containing Phthalonitrile Blends

**Figure 2:**
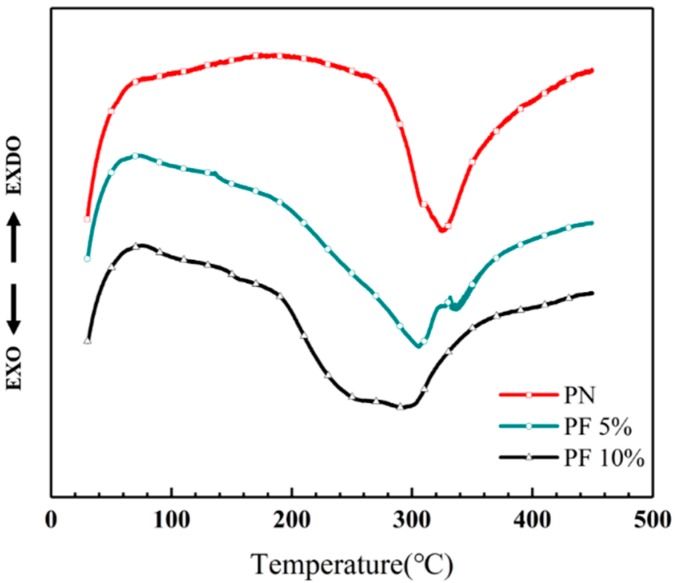
Differential scanning calorimetry (DSC) curves of novolac/phenol-containing phthalonitrile blends.

**Figure 3:**
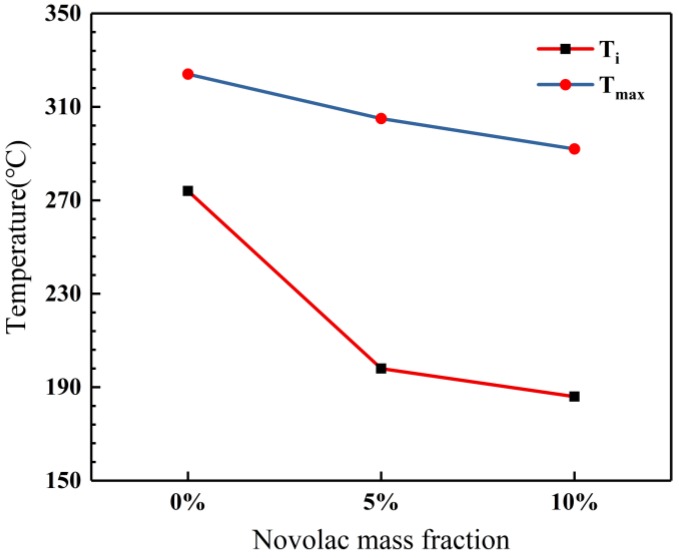
Influence of novolac mass percent on initial and maximum cure temperatures.

The novolac/phenol-containing phthalonitrile blends with different mass percent of novolac were investigated by differential scanning calorimeter, as shown in Figure 2. The DSC thermogram exhibits that all the blends only have an obvious exothermic peak corresponding to the polymerization reaction, which signifies that the blending between novolac and phthalonitrile was successful. Furthermore, it is observed that the peaks shift to lower temperature with the increase of novolac contents, which indicates that novolac could efficiently promote the crosslinking of nitrile group. The maximum cure temperature (Tmax) ranges from 292 to 324 °C for the different systems, and the blend containing the highest novolac content (10 wt%) exhibits an initial cure temperature (Ti) of 186 °C, compared with an initial cure temperature of 274 °C for phthalonitrile without novolac (Figure 3).

In Figure 2, a double peak can be clearly observed in the curve of PF 5% that is caused by the change of hydroxyl group concentration. In previous study, Nair [37] pointed out the content of hydroxyl group have an important influence on the curing mechanism of phenol-containing phthalonitrile resin. As the ratio of phenol to phthalonitrile groups grows from small to large, the formation of the product also goes form triazine and phthalocyanine to isoindoline. When the content of novolac is 5 wt%, the two modes of formation coexisted, resulting in a double peak in curve. Although the mass fraction of novolac addition increased to 10%, the mode of generating isoindoline dominated, and the two peaks tend to merge as a whole, which causes the double peak converts into a wide platform.

Meanwhile, the enthalpy of curing reaction can be obtained by integrating the exothermic peak, which indicates that the polymerization is getting vigorous with the raising mass percent of novolac. The enthalpy of PN is 25.06 J/g, which is significantly increased to 34.07 J/g with only 5 wt% addition of novolac. As the novolac content increase to 10 wt%, the reaction became more intense. Although the change of enthalpy is modest, the value still increases to 36.28 J/g. All the above phenomena can be explained by the catalysis of phenolic hydroxyl in the cyclization reaction of nitrile group. The curing properties data of novolac/phenol-containing phthalonitrile blends include enthalpy, Ti and Tmax is shown in Table 2.

**Figure 4:**
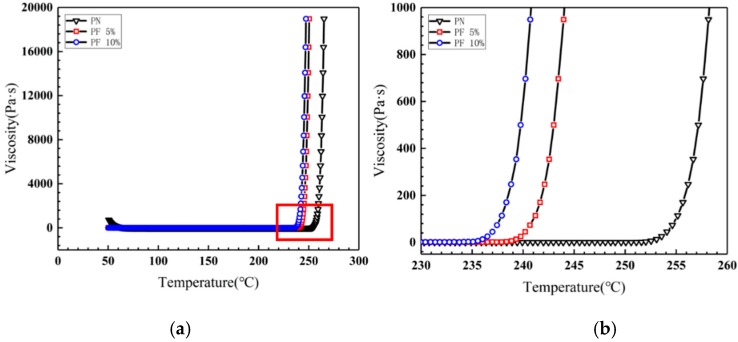
Viscosity vs. temperature plots for novolac/phenol-containing phthalonitrile blends: (**a**) viscosity vs. temperature cures at variable temperature from 50 °C to 300 °C; (**b**) local magnification of viscosity vs. temperature cures from 230 °C to 260 °C.

**Figure 5:**
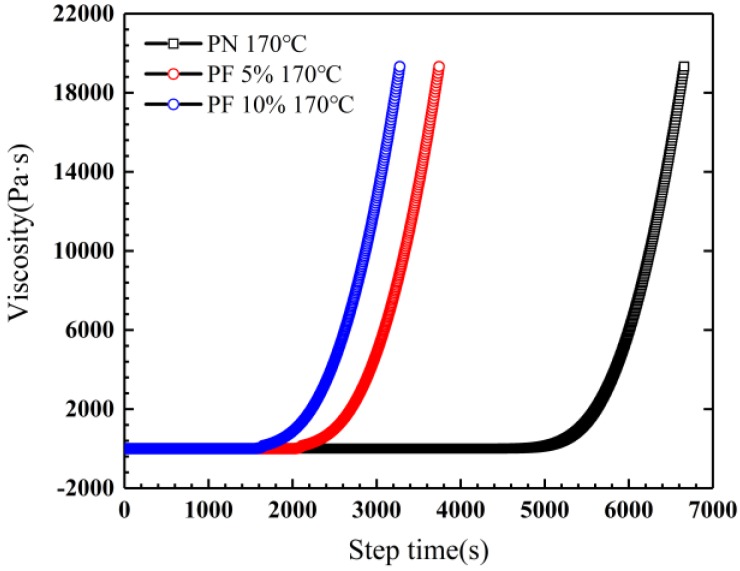
Isothermal rheological experiments for novolac/phenol-containing phthalonitrile blends at 170 °C.

Rheological experiments were conducted for novolac/phenol-containing phthalonitrile blends to compare the processability of the variation with different novolac contents, as shown in Figure 4. The blends all demonstrate outstanding processability, namely, the minimum viscosity less than 0.1 Pa·s and a widened processing window at ~200 °C. Note that the gelling point rapidly decreased when the novolac was added; it is consistent with the result of DSC test due to the phenolic hydroxyl catalysis. However, with the increase of novolac content, the downward trend of gelling point gets slower, of which the possible reason could be that the content of phenolic hydroxyl group is close saturation. Isothermal rheological experiments were performed at 170 °C to obtain the gelation time. As shown in Figure 5, the gelation time of the blend with 10 wt% novolac at 170 °C is 1641 s, 63.20% less than that of neat phthalonitrile (4461 s), showing that the blending improves the processability of phthalonitrile effectively.

### 3.2. Structure Transformation of Novolac/Phenol-Containing Phthalonitrile Copolymers

**Figure 6:**
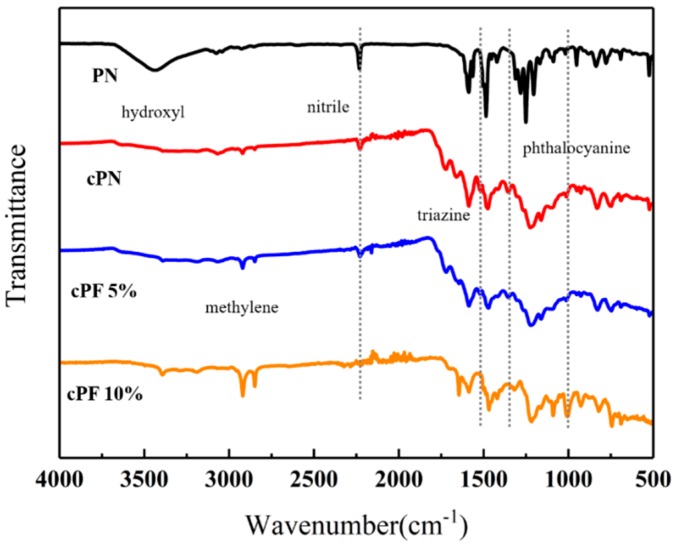
FT-IR spectra of phenol-containing phthalonitrile monomer and novolac/phenol-containing phthalonitrile copolymers.

**Scheme 1:**
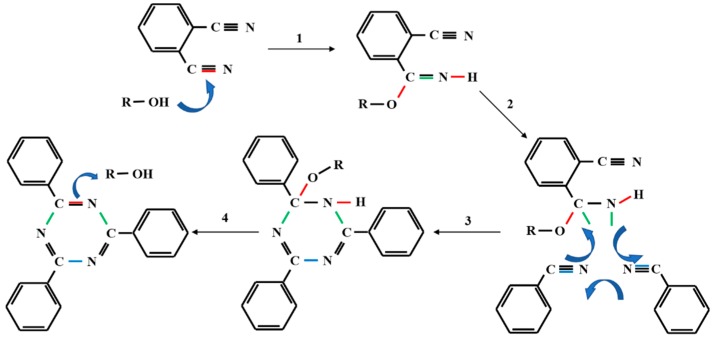
The mechanism to form the triazine ring catalyzed by phenolic hydroxyl group [37].

The structure of phenol-containing phthalonitrile monomer and novolac/phenol-containing phthalonitrile copolymers with different novolac contents was confirmed by FT-IR spectra, as shown in Figure 6. The phenol-containing phthalonitrile monomer contains the phenolic hydroxyl and nitrile groups corresponding to the absorption peak at 3400 cm^−1^ and 2233 cm^−1^, respectively. The analysis of copolymer without novolac presents the exhaustion of the hydroxyl group, and the significant diminution of nitrile absorption peak. However, the nitrile vibration peak did not disappear, which means that the reaction was not complete. The difficulty in obtaining complete curing is attributed to hydroxyl deficiency and space steric hindrance. After adding 5 wt% novolac, the absorption peak of nitrile declined. When the mass percent of novolac reaches 10%, the reaction of nitrile was almost complete while the hydroxyl was surplus. All the copolymers were in possession of triazine characteristic peak at 1520 cm^−1^ and 1360 cm^−1^ and vibration peak at 1010 cm^−1^ owing to phthalocyanine ring. Additionally, the methylene group with the absorption peaks at 2962 cm^−1^ and 2853 cm^−1^ was generated in the reaction. The mechanism to form the triazine ring catalyzed by phenolic hydroxyl group is shown in Scheme 1 [37].

### 3.3. Thermal Oxygen Stability of Novolac/Phenol-Containing Phthalonitrile Copolymers

**Figure 7:**
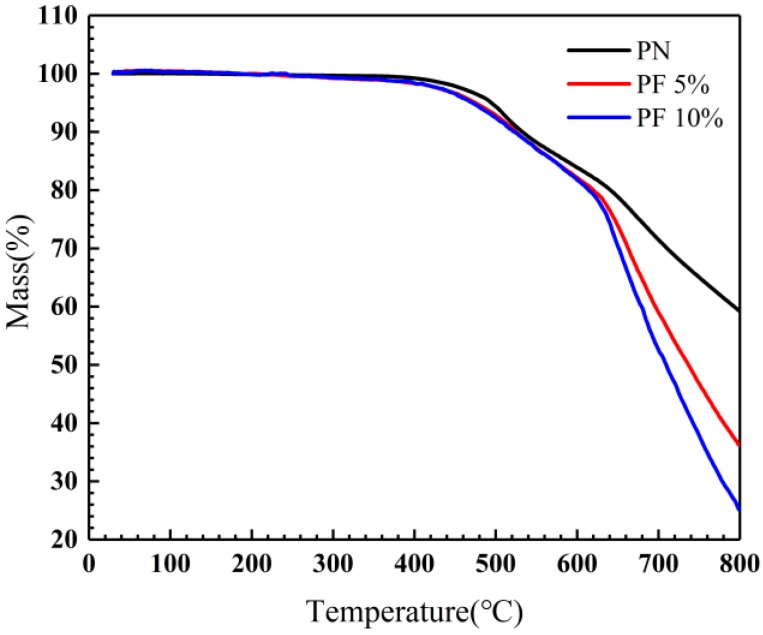
TGA of novolac/phenol-containing phthalonitrile copolymers in air.

The TGA was adopted to evaluate the influence of novolac content on the thermal oxygen stability of the novolac/phenol-containing phthalonitrile copolymers. The results of TGA show that all the copolymers reveal remarkable thermal oxygen stability. As shown in Figure 7, it is evident that the difference of mass loss between copolymers is tiny under 400 °C. At higher temperature ranging from 400 °C to 600 °C, the differences began to emerge. The residual mass of copolymers with novolac are a little less than that of neat phthalonitrile polymers, of which the possible reason was due to the decomposition of small molecules and parts of unreacted novolac resins. T_5%_ of 10 wt% novolac/phenol-containing phthalonitrile copolymers is 471 °C, close to 5 wt% novolac/phenol-containing phthalonitrile copolymers, approximate 20 °C lower than that of neat phenol-containing phthalonitrile polymers (494 °C). A significant difference of the weightlessness trend of copolymers was observed when the temperature goes above 600 °C. To be specific, residual mass of copolymers containing novolac declined rapidly, whereas the neat phenol-containing phthalonitrile polymers decomposed relatively slowly. The char yield at 800 °C of 10 wt% novolac was 25.01%, largely lower than that of the neat polymers (59.28%), revealing the massive decomposition of novolac and cross-linked network. In phenol-rich systems, phenol-mediated reaction of nitrile groups will be resulting in the formation of isoindoline groups [37]. The thermal stability of isoindoline is poorer than those of triazine and phthalocyanine groups formed in phthalonitrile-rich systems. Such a difference in thermal stability leads to significant difference in char yield between copolymers. The data of thermal oxygen stability for all investigated copolymers were summarized in Table 3.

### 3.4. Flexural Properties of Novolac/Phenol-Containing Phthalonitrile-Carbon Fabric Composites

**Figure 8:**
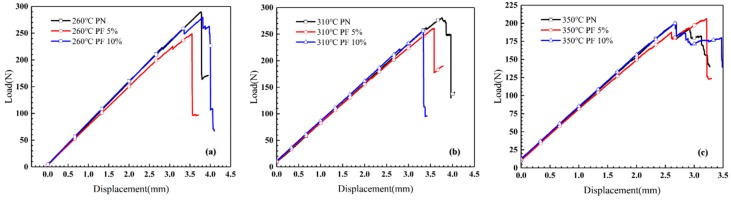
Load–displacement curves of composites bending test with different post-curing temperature and novolac mass percent: (**a**) 260 °C, (**b**) 310 °C, and (**c**) 350 °C.

**Figure 9:**
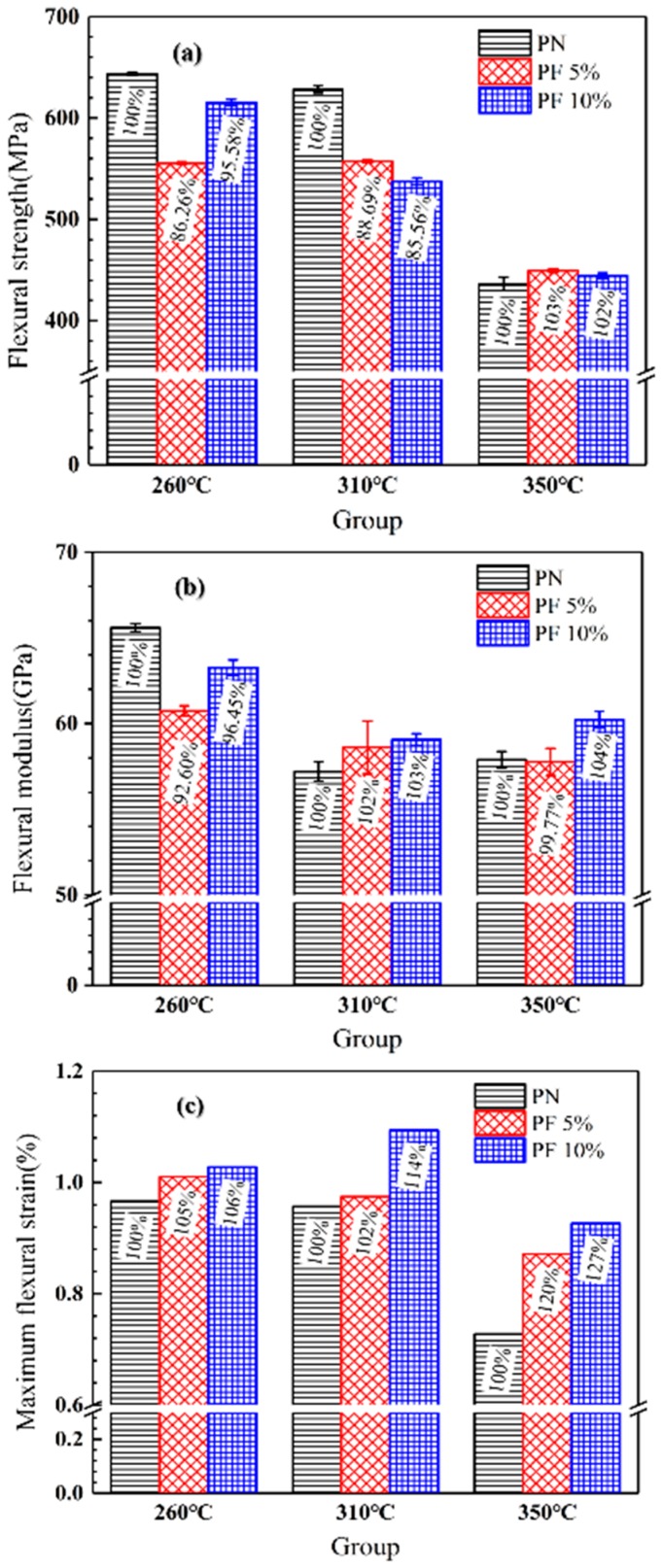
Flexural properties of novolac/phenol-containing phthalonitrile composites: (**a**) flexural strength, (**b**) flexural modulus, and (**c**) maximum flexural strain.

A three-point bending test of novolac/phenol-containing phthalonitrile laminates with different post-curing temperature and novolac mass percent was carried out. The typical load–displacement curves are shown in Figure 8, and the flexural properties including flexural strength, flexural modulus and maximum flexural strain are summarized in Figure 9. The data of flexural strength of the composites with different novolac mass percent shows a remarkable dispersion, but it can be observed that the flexural strength of the laminates post cured at 350 °C sharply declined to less than 450 MPa. The elevated post-curing temperature reveals negative effect on composites flexural strength regardless of novolac addition, which is attributed to oxidative decomposition of polymers. Nevertheless, the result of flexural strength in this paper is still higher than the reported 400 MPa [37]. As opposed to a drastic change in flexural strength, the flexural modulus is stable at around 60 GPa. The most prominent characteristic of flexural properties is the enhancement of elongation at bending. For those novolac/phenol-containing phthalonitrile composites post-cured at 350 °C, the maximum flexural strain of 10 wt% novolac addition is 0.93%, 27.35% higher than that of neat phenol-containing phthalonitrile composites (0.73%).

The purpose of novolac addition is to improve the toughness of phthalonitrile polymers with the assistance of the flexibility of phenolic segment. The objective is fulfilled due to the increase of the flexural elongation. However, the improvement is limited due to the fact that the flexural properties depend not solely on the toughness of the resins, but also on the interface properties between resins and reinforcement fiber.

### 3.5. Tensile Properties of Novolac/Phenol-Containing Phthalonitrile-Carbon Fabric Composites

**Figure 10:**
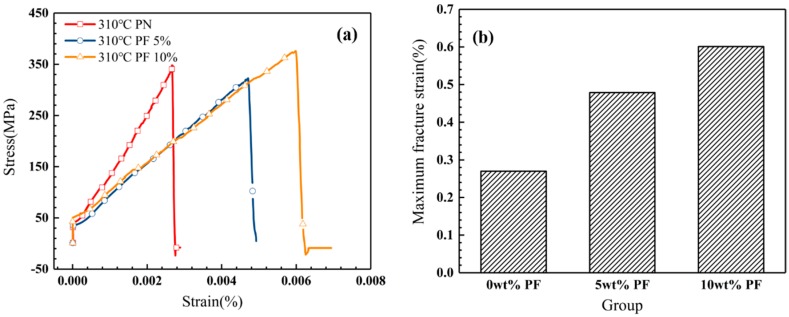
Tensile properties of novolac/phenol-containing phthalonitrile composites post curing at 310 °C: (**a**) stress–strain curves and (**b**) maximum fracture strain with different mass percent of novolac.

The uniaxial tensile experiment was adopted to verify the toughening effect of novolac addition in novolac/phenol-containing phthalonitrile composite systems. Different from the ambiguity of three-point bending test results, tensile experiment provides a forceful support to the purpose of novolac addition. Take the novolac/phenol-containing phthalonitrile-carbon fiber fabric composites post cured at 310 °C as an example, the maximum fracture strain of that composite with 10 wt% novolac is 0.60%, 122% higher than that of the composite without novolac addition (0.27%). The stress–strain curves of composites post cured at 310 °C is shown in Figure 10a and the maximum fracture strain is shown in Figure 10b.

**Figure 11:**
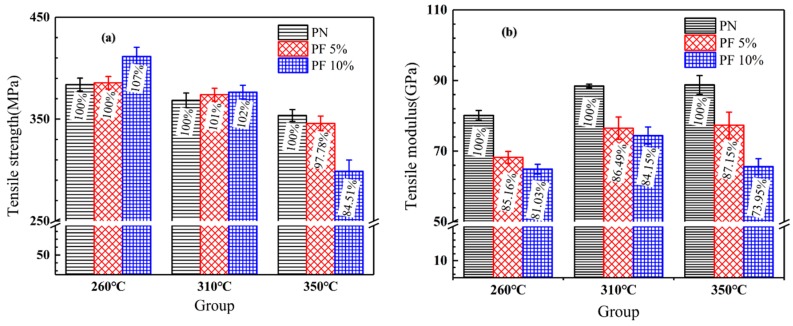
Tensile properties of novolac/phenol-containing phthalonitrile composites: (**a**) tensile strength; (**b**) tensile modulus.

The tensile strength and tensile modulus of novolac/phenol-containing phthalonitrile composites with different post-curing process are shown in Figure 11. It can be seen from the distribution of tensile strength that the increase of novolac mass percent enhances the strength of composites not post-cured or post-cured at a lower temperature of 310 °C. Meanwhile, with the change of novolac content, a sharply decline of the tensile modulus, independent from post-curing progress, is observed. In the research of curing properties of novolac/phenol-containing phthalonitrile blends covered in the previous section of this article, the curing degree of phthalonitrile enhanced with the addition of novolac which cause the improvement of composites tensile strength. On the other hand, from the fact that the tensile modulus reduces markedly, it can be asserted that the introduction of flexible phenolic segment improves the toughness of phthalonitrile resin. However, the tensile strength of composite post cured at 350 °C decrease significantly with the increase of novolac content, among which the most serious drop is the group with 10 wt% novolac content, due to the oxidative decomposition of resins.

### 3.6. Morphological Analysis

**Figure 12:**
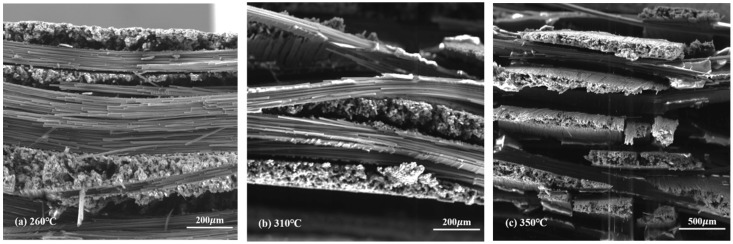
SEM micrographs of the tensile fractured surface of novolac/phenol-containing phthalonitrile composites with 5 wt% novolac: (**a**) 260 °C (×200), (**b**) 310 °C (×200), and (**c**) 350 °C (×100).

**Figure 13:**
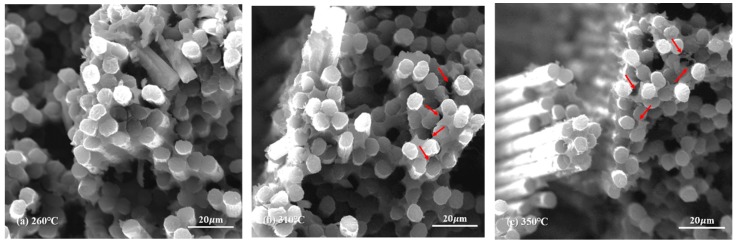
SEM micrographs of the tensile fractured surface of novolac/phenol-containing phthalonitrile composites with 5 wt% novolac: (**a**) 260 °C (×2000), (**b**) 310 °C (×2000), and (**c**) 350 °C (×2000).

The morphology of novolac/phenol-containing phthalonitrile composites was studied using fractured surfaces in tensile and flexural experiments. The SEM micrographs of tensile specimens which were post cured at different temperature are shown in Figure 12, and the high magnification images of that are shown in Figure 13. It can be seen from the low magnification photographs that the carbon fiber fabric was well wetted by resins. Although the post-curing temperature was elevated to 350 °C, the interface damage emerged, namely, the layers in composite were separated from each other, as shown in Figure 12c. Magnifying the power of the SEM, the single fiber was covered by resins tightly and the fracture end of carbon fibers were smooth, which can be clearly observed. However, as shown in Figure 13c, when the composite was post cured at elevated temperature, the existence of pores and cracks between fiber and resins can be observed. These defects were the main reasons that affected the mechanical properties of composites, leading to the decrease of tensile strength.

**Figure 14:**
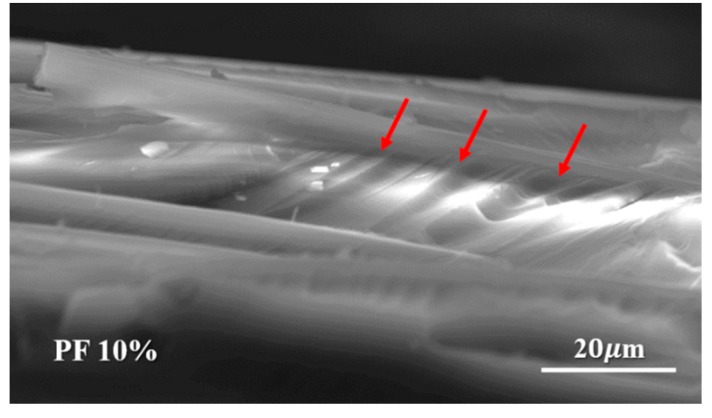
SEM micrographs of the flexural fractured surface of novolac/phenol-containing phthalonitrile composites with 10 wt% novolac.

The fractural surfaces of flexural specimens of novolac/phenol-containing phthalonitrile composites with different novolac content were investigated using scanning electron microscope, as shown in Figure 14. It can be seen that the fracture surface of composite with 10 wt% novolac was rough and a zig-zag crack propagation path was obvious, compared with phthalonitrile composite. It is well known that the fracture energy is consumed during the propagation of cracks. Therefore, the propagation of the zig-zag crack needs more energy than that of the linearity crack. The phenolic had flexible segments which could enhance the toughness of phthalonitrile resins, leading to the generation of the zig-zag crack, thus improving the mechanical properties of novolac/phenol-containing phthalonitrile composites. That is consistent with the results of mechanical properties investigation.

### 3.7. Dynamic Mechanical Analysis (DMA) of Novolac/Phenol-Containing Phthalonitrile Composites

**Figure 15:**
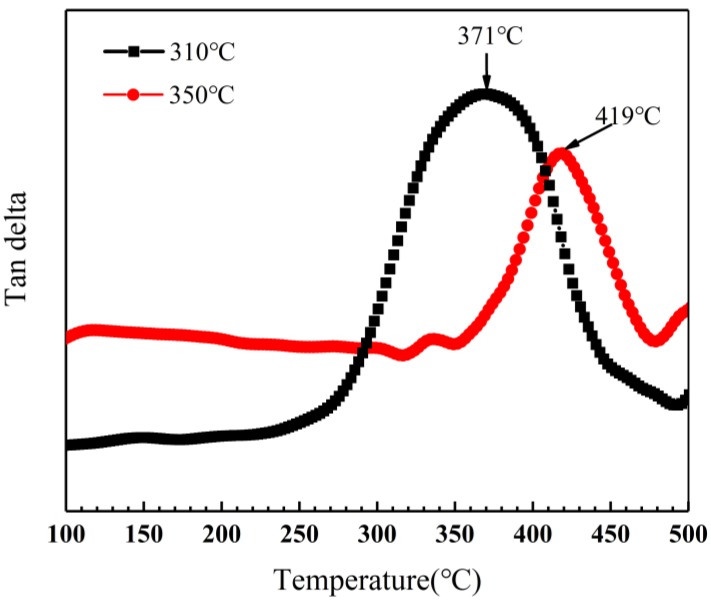
Loss tangent angle curves of phenol-containing phthalonitrile composites in different post-curing conditions.

Dynamic mechanical analysis was performed to determine the glass transition temperature of novolac/phenol-containing phthalonitrile composites with varied conditions. The loss tangent angle curves of composites without novolac addition are shown in Figure 15. With the diverse post-curing progress, the glass transition temperature of composites is 371 °C and 419 °C, respectively, according to the peak in the loss tangent angle curves. It is a remarkable promotion caused by elevated post-curing temperature agreeing with the consequences of mechanical properties due to the enhancement of curing degree.

**Figure 16:**
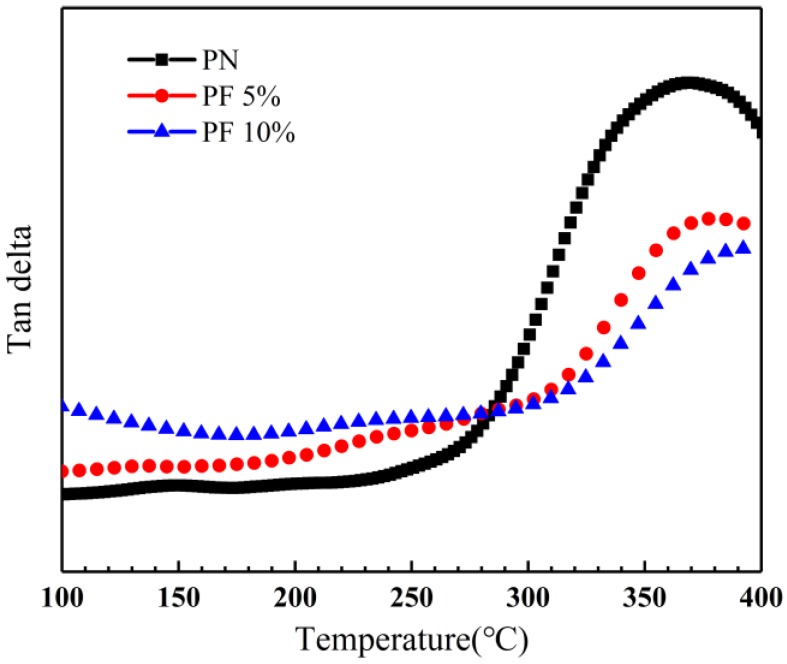
Loss tangent angle curves of novolac/phenol-containing phthalonitrile composites post-curing at 310 °C with different novolac content.

To identify the effect of novolac content on glass transition temperature, DMA of novolac/phenol-containing phthalonitrile composites with different novolac mass percent was applied, and the loss tangent angle curves is shown in Figure 16. The DMA specimens in Figure 14 were all post cured at 310 °C to control variables. The results show that the glass transition temperature increases with the augment of novolac mass percent, verifying the correlation between curing degree and novolac addition.

## 4. Conclusions

The novolac/phenol-containing phthalonitrile blends and composites were prepared and investigated. The curing properties of blends was discussed by DSC and rheological investments, revealing that the addition of novolac could efficiently promote the processability of blends, which manifested as the decrease of curing temperature and widening of processing window, which was related to the novolac content. FT-IR results showed that the conversion of nitrile group and curing degree was enhanced by novolac. However, the introduction of novolac will affect the thermal oxygen stability at elevated temperature over 600 °C dues to the decomposition of novolac and phenolic segment.

Research on composites materials shows that the flexible phenolic segment improves the toughness of phthalonitrile resin, leading to the remarkable promotion in flexural and tensile properties. In particular, the maximum fracture strain of composites with 10 wt% novolac during tensile tests 122% higher than that of the composite without novolac addition. Besides, it was also observed that the strength sharply decreased after post cured at an elevated temperature of 350 °C caused by high temperature oxidation damage. The morphological analysis also reveals that the defects caused by the elevated post-curing temperature, leading to a decrease of mechanical properties. The zig-zag crack was observed in fracture surface of composite with 10 wt% novolac, as an evidence of toughness improvement. Finally, the glass transition temperature of composites was confirmed to be elevated by the high post-curing temperature and high novolac content.

## Figures and Tables

**Table 1:** The curing schedule of the novolac/phenol-containing phthalonitrile–carbon fiber fabric composites.

Samples	Temperature, Pressure and Time
A	200 °C, 0.1 MPa, 20 min; 200 °C, 1.2 MPa, 1 h; 260 °C, 5 MPa,4 h
B	200 °C, 0.1 MPa, 20 min; 200 °C, 1.2 MPa, 1 h; 260 °C, 5 MPa,4 h; 310 °C, 5 MPa, 5 h
C	200 °C, 0.1 MPa, 20 min; 200 °C, 1.2 MPa, 1 h; 260 °C, 5 MPa,4 h; 350 °C, 5 MPa, 5 h

**Table 2:** Curing properties data of novolac/phenol-containing phthalonitrile blends.

Samples	Enthalpy (J/g)	Ti (°C)	Tmax (°C)
PN	25.06	274	324
PF 5%	34.07	198	305
PF 10%	36.28	186	292

**Table 3:** Thermal oxygen stability data of novolac/phenol-containing phthalonitrile copolymers.

Copolymers	T_5%_ (°C)	T_10%_ (°C)	Yc (%)
PN	495	533	59.28
PF 5%	475	525	36.27
PF 10%	471	523	25.01

T_5%_: Decomposition temperature at 5% mass loss; T_10%_: Decomposition temperature at 10% mass loss; Yc: Char yield at 800 °C.
